# Effect of fish paste products “Hanpen” intake in Sprague‐Dawley rats

**DOI:** 10.1002/fsn3.1569

**Published:** 2020-04-19

**Authors:** Kazunari Kadokura, Tsuyoshi Tomita, Masakazu Kobayashi, Tadahiko Mitsui, Kohei Suruga

**Affiliations:** ^1^ Food Function Research & Development Division International Operation Department Kibun Foods Inc. Inagi Tokyo Japan; ^2^ International Operation Department Kibun Foods Inc. Minato‐ku Tokyo Japan; ^3^ Purchasing Department Kibun Foods Inc. Minato‐ku Tokyo Japan

**Keywords:** atherogenic index, fish paste products, hanpen, HDL cholesterol, liver function, rats

## Abstract

Fish paste product, “Hanpen,” is a traditional type of Japanese food made from minced fish as well as imitation crab and kamaboko, and a marshmallow‐like soft texture is characteristic of hanpen. Hanpen is known as a high‐protein and low‐fat food. However, there is a lack of evidence on its health benefits. The aim of this study was to investigate the effects of hanpen intake on organ weight and biomarker levels in Sprague‐Dawley rats with diets consisting of hanpen for 84 days as an initial study. Male, 6‐week‐old Sprague‐Dawley rats were divided into two groups: group I, fed normal diets, and group II, fed normal diets with 5% dried hanpen. Throughout the 84‐day treatment period, we checked body weight and food intake, and after 84 days, we performed organ weight and blood biochemical analyses. No significant differences were seen in body weight, food intake, organ weight, and most biochemical parameters between group I and group II. Interestingly, total cholesterol (T‐CHO) and high‐density lipoprotein cholesterol (HDL‐C) levels of group II were significantly higher than those of group I after administration for 84 days. Moreover, lactate dehydrogenase (LDH) level of group II was marked lower than that of group I, and other liver function parameters of group II tended to be lower than those of group I. As conclusion, “Hanpen,” a Japanese traditional food, could be effective as a functional food for human health management worldwide.

## Graphical abstract

This is the first report about effect of fish paste products "Hanpen" intake in rats. “Hanpen,” a Japanese traditional food, could be effective as a functional food for human health management worldwide.

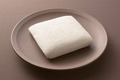

## 1 INTRODUCTION

Hanpen is a traditional type of fish paste product made from minced fish (surimi) in Japan like imitation crab and kamaboko (fish cake). Hanpen is prepared from many kinds of fish species such as pollack, threadfin beam, white croaker, red bigeye, blue shark, and pike eel (Kuronuma & Shimomura, 2019). In addition to minced fish, Japanese yam, egg white, starch, and salt are also key ingredients of hanpen, and it contains a lot of air by trapping large amounts of fine foam inside. Therefore, one major characteristic of hanpen is its marshmallow‐like, soft texture unlike imitation crab and kamaboko (Wakamatsu, Numata, & Nakamura, 1997). Generally in Japan, hanpen is known as a high‐protein and low‐fat food. However, very little research is available in the literature on health benefits of hanpen.

**FIGURE 1:**
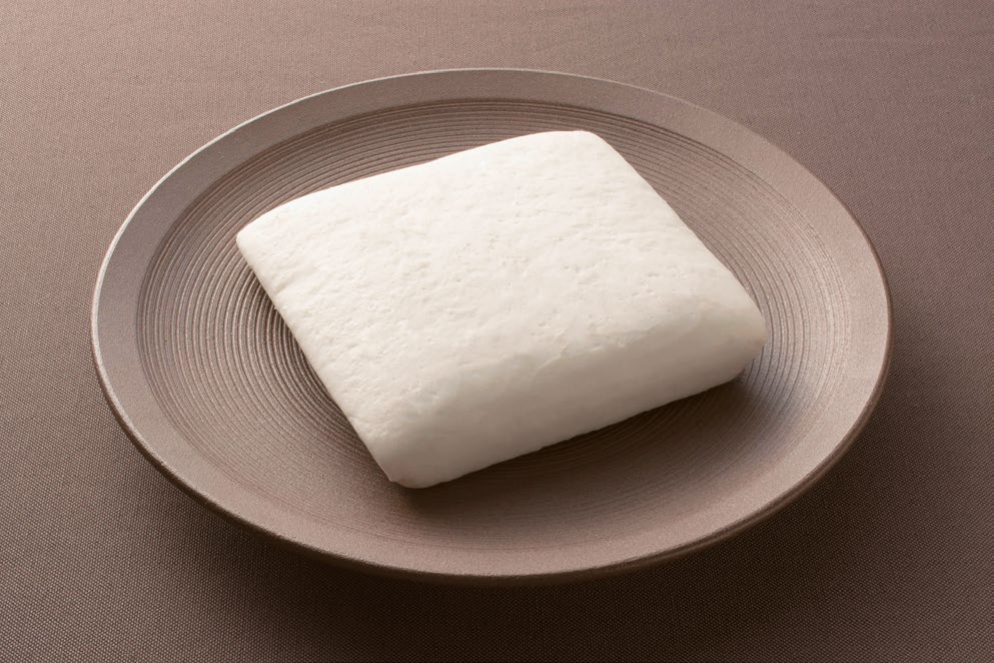
Fish paste product, KIBUN hanpen

Some fishes and fish paste products have bioactive compounds such as eicosapentaenoic acid (EPA), docosahexaenoic acid (DHA), and fish protein beneficial for human health. Arai, Kim, Chiba, and Matsumoto (2009) showed that fish oils containing EPA and DHA inhibited body weight gain and exhibited an anti‐obesity effect in female KK mice. Hung et al. (2000) reported that serum cholesterol (CHO), triglyceride (TG), and phospholipid levels of Sprague‐Dawley rats fed EPA or DHA for 3 weeks were significantly lower than those in the rats fed safflower oil. In addition, one study demonstrated that fish protein hydrolysate reduced plasma T‐CHO and increased the proportion of HDL‐C in male Wistar rats (Wergedahl et al., 2004). Moreover, Mizushige et al. (2010) investigated the effect of Alaska pollack protein (APP) intake with high‐fat diet on rats for 4 weeks and reported that intake of APP decreased serum TG and inhibited visceral body fat accumulation in rats. However, there are no experimental data on the effect of fish paste product, hanpen (Figure 1) intake in rats for 3 months.

In this study, we demonstrated the effect of hanpen intake on organ weight and biomarker levels in Sprague‐Dawley rats fed a diet comprising hanpen for 84 days for the first time.

## 2 MATERIALS AND METHODS

### 2.1 Materials

The commercial KIBUN hanpen (Figure 1) was lyophilized. In brief, minced fish, surimi (pollack, red bigeye, and shortfin shark), were ground with Japanese yam, egg white, starch, salt, and other ingredients; trapped large amounts of fine foam inside; and then boiled.

### 2.2 Animal experiments

Male, 6‐week‐old Sprague‐Dawley rats purchased from SLC Japan, Inc. (Shizuoka, Japan) were individually housed under a 12‐hr light/dark cycle (light phase; 8 a.m.‐8 p.m., dark phase; 8 p.m.‐8 a.m.) at a temperature of 23 ± 2°C, relative humidity of 50 ± 20% with aseptic food and tap water ad libitum, and divided into two groups: group I, fed AIN‐93G (*n* = 8), and group II, fed AIN‐93G with 5% dried hanpen (*n* = 8). Subjects had free access to food for 84 days. Body weight and food intake were measured once a week for each rat. After 84 days of administration, all rats were sacrificed under isoflurane inhalation anesthesia (concentration for induction of anesthesia: 4%, concentration for maintenance: 2%), and blood corresponding to a nonfasting state was collected. The blood was centrifuged at 1,657 *g* at 4°C for 10 min and stored at −80°C until the analysis. The weight of liver, spleen, kidney, white adipose tissues, interscapular brown adipose tissue, and skeletal muscle of each rat were measured. The experiments were performed at LA Centre in Oriental Yeast Co., Ltd. and authorized by the Japanese Government. The present study was conducted according to the ethical guidelines for laboratory animals and the standard operating procedures of the laboratory. The experimental protocol was approved by the animal experiment ethics committee of the laboratory (approval no. 19,003).

### 2.3 Diets

Rats in group I were fed the AIN‐93G (Oriental Yeast Co., Ltd.) as control diet. Rats in group II were given a diet in which dried hanpen replaced casein, L‐cystine, and β‐cornstarch. Formulation and nutrients of experimental diets in this study are shown in Tables 1 and 2.

**TABLE 1:** Composition of the experimental diet

	Group I (without hanpen)	Group II (with hanpen)
Casein	20.0	18.0
L‐Cystine	0.30	0.27
β‐Cornstarch	39.7486	36.0786
α‐Cornstarch	13.2	13.2
Sucrose	10.0	10.0
Soybean oil	7.0	7.0
Cellulose	5.0	5.0
Mineral mix, AIN−93G‐MX	3.5	—
Modified mineral mix	—	3.5
Tripotassium citrate	—	0.7
Vitamin mix, AIN−93G‐VX	1.0	1.0
Choline bitartrate	0.25	0.25
Butylhydroquinone	0.0014	0.0014
Hanpen dry powder	—	5.0
Total (%)	100	100

**TABLE 2:** Composition of the nutritional analysis of the experimental diet

		Group I (without hanpen)	Group II (with hanpen)
Water	g	9.00	9.00
Energy	kcal	368.00	368.70
Crude protein	g	18.10	18.10
Crude fat	g	7.30	7.20
Crude ash	g	3.10	2.90
Crude fiber	g	5.00	5.00
Nitrogen‐free extract	g	57.60	57.80
Ca	g	0.52	0.52
P	g	0.32	0.32
Mg	g	0.05	0.05
Na	g	0.10	0.11
K	g	0.36	0.35

Abbreviations: Ca, calcium; K, potassium; Mg, magnesium; Na, sodium; P, phosphorus.

### 2.4 Statistical analysis

The results are presented as mean ± standard error. Statistical significance was evaluated by Student's *t* test. A *p*‐value of less than .05 was considered statistically significant.

## 3 RESULTS

### 3.1 Effect of hanpen on body weight, organ weight, adipose tissue weight, and muscle weight in Sprague‐Dawley rats

The effects of oral administration of AIN‐93G with 5% hanpen on body weight, organ weight, adipose tissue weight, and muscle weight in Sprague‐Dawley rats are shown in Table 3. Total food intake of group II (1,906 ± 41 g for 84 days) tended to be higher than those of group I (1,765 ± 53 g for 84 days), but no significant differences were observed. Differences in body weight, spleen weight, adipose tissue weight, and muscle weight between group I and group II were not significant after administration for 84 days. On the contrary, liver and kidney weights of group II (liver: 22 ± 0.9 g, kidney: 3.5 ± 0.1 g) were higher than those of group I (liver: 19 ± 0.9 g, kidney: 3.1 ± 0.1 g) after 84 days, respectively.

**TABLE 3:** Effect of hanpen on body weight, organ weight, adipose tissue weight, and muscle weight in Sprague‐Dawley rats (*n* = 8)

		Group I (without hanpen)	Group II (with hanpen)
Total food intake	g	1765.230 ± 53.057	1906.075 ± 41.148
Initial body weight	g	193.788 ± 1.687	193.663 ± 2.651
Final body weight	g	559.138 ± 18.433	589.038 ± 12.179
Liver	g	19.335 ± 0.869	22.245 ± 0.851*, a, **
Kidney	g	3.119 ± 0.088	3.480 ± 0.106*, a, **
Spleen	g	0.902 ± 0.048	0.908 ± 0.040
White adipose tissue	g	27.660 ± 2.190	30.299 ± 1.065
Brown adipose tissue	g	0.291 ± 0.020	0.285 ± 0.021
Muscle	g	6.960 ± 0.217	6.556 ± 0.066

Note

Results are expressed as mean ± standard error. Statistical significance was evaluated by Student's *t* test.

* *p* < .05 versus group I.

### 3.2 Effect of hanpen on biochemical parameters in Sprague‐Dawley rats

Table 4 shows the analysis of blood biochemical parameters of rats after diets containing 5% dried hanpen administration. No marked differences were seen in almost all the biochemical parameters between group I and group II. The inorganic phosphorus (IP) level of group II (5.8 ± 0.1 mg/dl) was higher than that of group I (4.8 ± 0.1 mg/dl). The T‐CHO and HDL‐C levels of group II (T‐CHO: 104 ± 5.4 mg/dl, HDL‐C: 34 ± 1.2 mg/dl) were higher than those of group I (T‐CHO: 83 ± 5.4 mg/dl, HDL‐C: 27 ± 1.6 mg/dl) after 84 days, respectively. Moreover, interestingly, the LDH level of group II (492 ± 69 IU/L) was significantly lower than that of group I (700 ± 46 IU/L).

**TABLE 4:** Effect of hanpen on blood biochemical parameters in Sprague‐Dawley rats after 84 days of administration

		0‐day administration (*n* = 3)	84‐day administration (*n* = 8)	
			Group I (without hanpen)	Group II (with hanpen)
TP	g/dL	5.533 ± 0.027	6.838 ± 0.025	6.975 ± 0.093
ALB	g/dL	4.000 ± 0.000	4.250 ± 0.050	4.363 ± 0.081
A/G	‐	2.633 ± 0.054	1.663 ± 0.053	1.675 ± 0.039
BUN	mg/dL	7.933 ± 0.307	17.463 ± 0.683	15.888 ± 0.421
CRE	mg/dL	0.210 ± 0.009	0.336 ± 0.012	0.350 ± 0.009
Na	mEq/L	141.667 ± 0.272	142.375 ± 0.246	142.750 ± 0.293
K	mEq/L	4.967 ± 0.191	4.238 ± 0.050	4.263 ± 0.043
Cl	mEq/L	97.333 ± 0.272	100.750 ± 0.342	100.250 ± 0.293
Ca	mg/dL	11.000 ± 0.082	10.625 ± 0.061	10.613 ± 0.054
IP	mg/dL	9.667 ± 0.136	4.763 ± 0.137	5.800 ± 0.113*, a, **
Fe	µg/dL	120.333 ± 3.193	158.375 ± 5.282	157.750 ± 3.896
AST	IU/L	67.000 ± 8.340	85.750 ± 7.271	70.250 ± 4.137
ALT	IU/L	30.333 ± 2.126	32.375 ± 2.744	31.875 ± 3.891
ALP	IU/L	1071.333 ± 80.656	469.375 ± 34.741	461.850 ± 25.766
LDH	IU/L	139.667 ± 5.041	699.500 ± 45.934	491.625 ± 69.099*
LAP	IU/L	47.000 ± 2.160	47.875 ± 0.648	46.375 ± 0.951
AMY	IU/L	2119.333 ± 81.198	2097.750 ± 68.254	2046.125 ± 60.536
γ‐GT	IU/L	3.000>	3.000>	3.000>
ChE	IU/L	5.000>	5.000>	5.000>
T‐CHO	mg/dL	70.375 ± 2.778	83.250 ± 5.431	103.750 ± 5.367*
F‐CHO	mg/dL	18.000 ± 0.471	20.875 ± 1.473	23.875 ± 1.634
E‐CHO	mg/dL	64.667 ± 3.538	77.000 ± 4.953	85.750 ± 4.670
E/T	%	78.000 ± 0.471	78.625 ± 0.660	78.375 ± 0.585
TG	mg/dL	74.125 ± 7.330	155.375 ± 24.576	132.500 ± 16.717
LDL‐C	mg/dL	7.667 ± 0.272	9.875 ± 0.799	9.125 ± 0.571
HDL‐C	mg/dL	29.750 ± 0.964	26.500 ± 1.649	34.000 ± 1.173*
AIP^*^, ^a^, ^**^	‐	0.022 ± 0.050	0.381 ± 0.065	0.205 ± 0.054
GLU	mg/dL	242.000 ± 10.677	176.250 ± 3.944	179.875 ± 5.357
T‐BIL	mg/dL	0.030 ± 0.005	0.046 ± 0.005	0.060 ± 0.004
D‐BIL	mg/dL	0.000 ± 0.000	0.004 ± 0.002	0.004 ± 0.004
I‐BIL	mg/dL	0.030 ± 0.005	0.043 ± 0.005	0.056 ± 0.004
TBA	µmol/L	12.667 ± 0.981	14.125 ± 2.281	9.625 ± 1.648

Note

Results are expressed as mean ± standard error. Statistical significance was evaluated by Student's *t* test.

Abbreviations: AIP, atherogenic index of plasma; ALB, albumin; ALP, alkaline phosphatase; ALT, alanine transaminase; AMY, amylase; AST, aspartate aminotransferase; BUN, blood urea nitrogen; Ca, calcium; ChE, cholinesterase; Cl, chloride; CRE, creatinine; D‐BIL, direct bilirubin; E‐CHO, esterified cholesterol; F‐CHO, free cholesterol; Fe, iron; GLU, glucose; HDL‐C, high‐density lipoprotein‐cholesterol; I‐BIL, indirect bilirubin; IP, inorganic phosphorus; K, potassium; LAP, leucine aminopeptidase; LDH, lactate dehydrogenase; LDL‐C, low‐density lipoprotein‐cholesterol; Na, sodium; TBA, total bile acids; T‐BIL, total bilirubin; T‐CHO, total cholesterol; TG, triglyceride; TP, total protein; γ‐GT, γ‐glutamyl transpeptidase.

^a^ AIP: log (TG/HDL‐C) with TG (mg/dL/88.57) and HDL‐C (mg/dL/38.67) expressed in mmol/L (Edwards, Blaha, & Loprinzi, 2017).

** *p* < .01 versus group I.

* *p* < .05 versus group I.

## 4 DISCUSSION

A traditional Japanese diet and Japanese food products are been widely known to be healthy, contributing to longevity and preventing various noncommunicable diseases (Gabriel, Ninomiya, & Uneyama, 2018; Ueshima, 2007). The traditional Japanese diets include rice, miso soup, soybean products, vegetables, fruits, Japanese pickles, seaweed, mushrooms, green tea, and fish (Kanauchi & Kanauchi, 2019). In addition to these products, fish paste products including kamaboko, imitation crab, and hanpen are also traditional Japanese foods. These products are made from minced fish, starch, and salt, and are easy to eat in comparison with raw fish containing skeletal bone and fish guts. Among them, hanpen, which contains Japanese yam and egg white as well as minced fish, starch, and salt, has a marshmallow‐like, soft texture by trapping large amounts of fine foam inside (Figure 1). Raw hanpen (KIBUN hanpen) contains approximately 10.3% protein and 0.2% fat, and it is known as a high‐protein and low‐fat food. However, few studies have been conducted on its health benefits. In this study, we aimed to investigate the effects of hanpen intake on organ weight and biomarker levels in Sprague‐Dawley rats for the first time. Male, 6‐week‐old Sprague‐Dawley rats were divided into two groups: group I, fed AIN‐93G, and group II, fed AIN‐93G with 5% dried hanpen.

No deaths or abnormalities in food consumption and coat condition were noted in the hanpen‐administered rats in this study. From the study results, we confirmed that the hanpen did not induce any adverse reaction in rats after 84 days of administration. No significant differences were found in body weight, organ weight, and most biochemical parameters between group I and group II because nutrition levels of group II diets were almost equal to those of group I diets (Table 2).

No significant differences in total food intake were found, although that of group II tended to be higher than that of group I. The taste of hanpen might be suitable to Sprague‐Dawley rats in comparison with normal diets. Liver and kidney weight gains in group II were observed unlike those of group I (*p* < .05) after administration for 84 days. Therefore, we calculated the relative organ weight to body weight (%) using formulae described by Vani and Reddy (2000).

$$\text{Relative organ to body weight}\;\left(\%\right)=\text{Actual weight of the organ}\left(g\right)/\text{Body weight}\left(g\right)\times 100$$

Relative organ to body weight of liver and kidney in group II was 3.77% and 0.59%, respectively, and no significant differences in those values were obtained when compared with group I (liver, 3.45%; kidney, 0.55%). There were no significant differences in the final body weight, spleen weight, and adipose tissue weight between group I and group II. Moreover, there were no differences in muscle weight between groups. The diets of group I, AIN‐93G, had 20% casein as main protein souse, and the diets of group II contained 5% dried hanpen instead of mainly casein. Generally, protein ingestion in the form of casein and whey increases the amino acid supply to muscles, which further promotes muscle protein synthesis Reidy et al. (2013). Dort et al. (2013), reported that cod protein induced anti‐inflammatory activity and had an effect on skeletal muscle repair after an injury in male Wistar rats. These studies and our findings indicate that the fish paste product, hanpen, may be a protein source for skeletal muscle synthesis as well as casein.

In the biomarker analysis, the IP level of group II was higher than that of group I (*p* < .01). Hatayama et al. (2002) investigated the background data of blood chemistry parameters in Crj:CD(*SD*)IGS rats and reported that standard value of IP in 10‐week‐old, 19‐week‐old, and 32‐week‐old rats is range of 7.7 ± 0.6, 6.5 ± 0.8, and 6.0 ± 0.9 mg/dl, respectively. From this study, we assumed that the IP level of group II is almost in rage of this standard value.

Hyperlipidemia is characterized by an increase in plasma T‐CHO, TG, low‐density lipoprotein cholesterol (LDL‐C), and/or decrease in HDL‐C (Wu et al., 2014). T‐CHO and HDL‐C levels of group II were significantly higher than that of group I after 84 days of administration. On the other hand, the TG and LDL‐C levels of group II tended to be lower than that of group I. Therefore, it was assumed that high T‐CHO levels of group II were predominantly related to HDL‐C levels increasing. From this result, it was considered that hanpen intake was effective in protecting hyperlipidemia. Further, the atherogenic index of plasma (AIP) in group II (0.205 ± 0.054, *p* = .07 vs. group I) was inclined to decrease in comparison with group I (0.381 ± 0.065). The serum TG levels and AIP are risk factors for coronary heart disease (NIH Consensus conference, 1993; Wu, Gao, Zheng, Ma, & Xie, 2018), and hanpen intake may be an effective protection against coronary heart disease. Dried KIBUN hanpen contains approximately 0.08% EPA and 0.22% DHA, and levels of omega‐3 fatty acids and omega‐6 fatty acids in dried hanpen are 0.33% and 0.07%, respectively. EPA and DHA are very long chains of omega‐3 fatty acids, and their daily intake is recommended by many global dietary guidelines (Sioen et al., 2017). Some studies reported the effect of EPA and/or DHA on lipid metabolism. For instance, there are reports that the intake of EPA lowers plasma TG in animal studies (Ding et al., 2016; Shang et al., 2017). Clinical studies that investigated EPA also demonstrated that it lowers TG and non‐HDL‐C levels (Ballantyne et al., 2012; Bays et al., 2011). In addition, Abdelhamid et al. (2018) reported in their review articles that EPA and DHA slightly reduced serum TG and raised HDL‐C. From these reports, it was presumed that the effects of group II including hanpen on lipid metabolism may be related to EPA and DHA.

Interestingly, LDH level of group II was significantly lower than that of group I and the AST level of group II tended to be lower than that of group I. In addition to LDH and AST levels, the values that related to liver function such as ALT, ALP, and LAP of group II were likely to decrease compared with that of group I. These data indicated that hanpen intake might be effective for preventing liver function deterioration. The polyunsaturated fatty acids including EPA and DHA have been recommended as the dietary strategy to protect against nonalcoholic fatty liver disease, because EPA and DHA induced high antioxidant, anti‐inflammatory, and hypolipidemic effects (Bays, Ballantyne, Braeckman, Stirtan, & Soni, 2013; Dangardt et al., 2010). One ingredient in hanpen is Japanese yam which contains diosgenin, a steroid saponin which has reduced doxorubicin (DOX)‐induced cardiotoxicity in mice (Kusano, Tsujihara, Masui, Kozai, & Takeuchi, 2016; Patel, Gadewar, Tahilyani, & Patel, 2012). Chen, Wang, Hsu, Lin, and Chen (2017) reported that yam (*Dioscorea japonica* Thunb.) extracts including diosgenin had an antioxidant and anti‐inflammatory effect, and contributed to increasing glutathione peroxidase and superoxide dismutase activities in DOX‐treated mice. We assumed that the values related to liver function of group II were lower than that of group I, because EPA, DHA, and Japanese yam including diosgenin were contained in KIBUN hanpen.

## 5 CONCLUSION

In summary, the Japanese traditional food, KIBUN hanpen, facilitated skeletal muscle synthesis as well as casein, raising plasma HDL‐C level, and prevention of liver function deterioration after 84 days of administration in Sprague‐Dawley rats. Hanpen is easy to eat in comparison with raw fish, which contains skeletal bone and fish guts, and is beneficial to human health. This study is a preliminary investigation of the effects of hanpen intake on organ weight and biomarker levels in rats. Thus, the mechanisms of hanpen on raising plasma HDL‐C level and prevention of liver function deterioration remain unclear. However, hanpen could be effective as a functional food for human health management worldwide.

## CONFLICT OF INTERESTS

The authors have no conflict of interest to report.

## AUTHOR CONTRIBUTION

KK, TT, and KS designed the study and conducted the experiments. KK, MK, TM, and KS prepared fish paste products hanpen. KK, TT, and KS analyzed the data and wrote the manuscript.

## ETHICAL STATEMENTS

The experiments were performed at LA Centre in Oriental Yeast Co., Ltd. and authorized by the Japanese Government. The present study was conducted according to the ethical guidelines for laboratory animals and the standard operating procedures of the laboratory. The experimental protocol was approved by the animal experiment ethics committee of the laboratory (approval no. 19003).
